# Peptidylarginine deiminases: novel drug targets for prevention of neuronal damage following hypoxic ischemic insult (HI) in neonates

**DOI:** 10.1111/jnc.12744

**Published:** 2014-05-24

**Authors:** Sigrun Lange, Eridan Rocha-Ferreira, Laura Thei, Priyanka Mawjee, Kate Bennett, Paul R Thompson, Venkataraman Subramanian, Anthony P Nicholas, Donald Peebles, Mariya Hristova, Gennadij Raivich

**Affiliations:** * UCL Institute for Women's Health, Maternal & Fetal Medicine, Perinatal Brain Repair GroupLondon, UK; † UCL School of PharmacyLondon, UK; ‡ UCL Institute for Women's Health, NeonatologyLondon, UK; § Department of Chemistry, The Scripps Research InstituteJupiter, Florida, USA; ¶ Department of Neurology, University of Alabama at Birmingham and Birmingham VA. Medical CenterBirmingham, Alabama, USA; ** UCL Medical SchoolLondon, UK

**Keywords:** hypoxic ischaemic encephalopathy, lipopolysaccharide, microglia, neonatal, peptidylarginine deiminases, protein deimination/citrullination

## Abstract

Neonatal hypoxic ischaemic (HI) injury frequently causes neural impairment in surviving infants. Our knowledge of the underlying molecular mechanisms is still limited. Protein deimination is a post-translational modification caused by Ca^+2^-regulated peptidylarginine deiminases (PADs), a group of five isozymes that display tissue-specific expression and different preference for target proteins. Protein deimination results in altered protein conformation and function of target proteins, and is associated with neurodegenerative diseases, gene regulation and autoimmunity. In this study, we used the neonatal HI and HI/infection [lipopolysaccharide (LPS) stimulation] murine models to investigate changes in protein deimination. Brains showed increases in deiminated proteins, cell death, activated microglia and neuronal loss in affected brain areas at 48 h after hypoxic ischaemic insult. Upon treatment with the pan-PAD inhibitor Cl-amidine, a significant reduction was seen in microglial activation, cell death and infarct size compared with control saline or LPS-treated animals. Deimination of histone 3, a target protein of the PAD4 isozyme, was increased in hippocampus and cortex specifically upon LPS stimulation and markedly reduced following Cl-amidine treatment. Here, we demonstrate a novel role for PAD enzymes in neural impairment in neonatal HI Encephalopathy, highlighting their role as promising new candidates for drug-directed intervention in neurotrauma.

Hypoxic Ischaemic Insult (HI) results in activation of peptidylarginine deiminases (PADs) because of calcium dysregulation. Target proteins undergo irreversible changes of protein bound arginine to citrulline, resulting in protein misfolding. Infection in synergy with HI causes up-regulation of TNFα, nuclear translocation of PAD4 and change in gene regulation as a result of histone deimination. Pharmacological PAD inhibition significantly reduced HI brain damage.

Brain injury around the time of birth is a major contributing factor to cerebral palsy and other neurological disabilities that affect one to three cases per 1000 births in the western world, and to a higher extent in less developed countries (Perlman 2006). Oxygen deprivation, and more recently, infection (Wu and Colford 2000; Hagberg *et al.* 2002; Mallard *et al.* 2003), have been identified as major causes of perinatal brain injury in term as well as preterm babies. On the experimental side, neonatal animal models have shown a role for epigenetic mechanisms (Kumral *et al.* 2012), pH changes (Robertson *et al.* 2004; Kendall *et al.* 2011a) as well as for the tumour necrosis factor (TNF) gene cluster of cytokines in the context of a combined inflammatory and hypoxic-ischaemic (HI) insult (Kendall *et al.* 2011b).

From a clinical perspective, several recent studies have clearly shown a moderate but significant protective effect of hypothermia (Wyatt *et al.* 2007). However, at present, treatment of severe cases of HI Encephalopathy (HIE) is still rather limited. Therefore, novel or adjunct treatments, which would enhance post-insult neuroprotection beyond what is observed with hypothermia alone, are of considerable interest. For example, recent translational studies have documented significantly enhanced neuroprotection following cotherapy of hypothermia with Xenon (Faulkner *et al.* 2011) or with melatonin (Robertson *et al.* 2013). The reoxygenation following a neonatal HI insult is frequently followed by an intermediate ‘grace period’ with little overt metabolic, NMR or histological abnormalities, and only then by secondary energy failure (Faulkner *et al.* 2011; Wyatt *et al.* 1989; Stys 1998) apoptotic, necrotic and/or autophagic cell death and axonal degeneration (Dragunow *et al.* 1993; Adhami *et al.* 2007). Changes in cellular transcription, *de novo* protein synthesis and post-translational chemical modification all play pivotal roles during this intermediate phase (Culman *et al.* 2007; Pirianov *et al.* 2007; Yi *et al.* 2007). Identifying novel key factors mediating white and grey matter damage will allow both better understanding of the mechanism of the injury process as well as facilitating clinical intervention.

Arginine deimination/citrullination is a post-translational modification mediated by Ca^+2^-activated peptidylarginine deiminases (PADs). Positively charged protein arginine residues are modified irreversibly into hydrophilic but uncharged citrullines on target proteins. This is distinct from processes that create free L-citrulline as an intermediate in the urea acid cycle or as a by-product of nitric oxide synthase reactivity (Keilhoff *et al.* 2008). The substitution of an imino- for oxy-group at the arginine guanidinium residue produces a loss of one positive charge and release of ammonia (Vossenaar *et al.* 2003). The incidental disruption of ionic and hydrogen bonds within the substrate proteins causes wide-ranging effects on structure and function of protein–protein interactions. The PADs comprise a group of five isozymes with tissue-specific expression and different preference for target proteins. PAD2 and PAD4 are regarded as the prominent isozymes in the CNS, but PAD3 expression has also been described in the CNS (Vossenaar *et al.* 2003; Gyorgy *et al.* 2006). Studies on neuronal and inducible nitric oxidase synthases, enzymes that convert free arginine to citrulline, have shown them not to be involved in enhanced peptidyl-citrulline immunosignalling (Keilhoff *et al.* 2008). Structures especially prone to protein deimination are β-turns and the intrinsically disordered proteins which are abundant in the CNS (Gyorgy *et al.* 2006). Some of the main targets identified are nuclear histones (Wang *et al.* 2004); structural proteins including components of the myelin sheath; intermediate filaments and associated adaptor proteins; extracellular components such as fibrin and fibronectin (Gyorgy *et al.* 2006; Lange *et al.* 2011), and the chemokines (Loos *et al.* 2008, 2009; Proost *et al.* 2008). Deimination affects upstream cytokines and chemokines such as TNFα and CXCL8 & 10 (Moelants *et al.* 2011, 2013). Apart from being involved in physiological processes during development (Hagiwara *et al.* 2002; Li *et al.* 2008; Horibata *et al.* 2012), protein deimination has been detected in many human inflammatory and degenerative diseases including multiple sclerosis, Alzheimer's dementia, Creutzfeldt–Jakob disease, glaucoma and rheumatoid arthritis (Moelants *et al.* 2013). Recently, Lange *et al*. (2011) demonstrated a novel functional role in spinal cord injury by using pharmacological inhibition of protein deimination with the pan-PAD inhibitor Cl-amidine. This resulted in significantly reduced cavity size, neuronal damage and apoptosis in the injured spinal cord. As this was the first, and so far sole study to show a role for PADs in neuronal injury, and protein deimination has not been described in brain injury before, our question was if the protective effect of PAD inhibition would be translatable to other models of neuronal damage. In the current study, we demonstrate a new functional role for the PADs in neuronal damage in the neonatal brain in response to standard HIE insult as well as in the infection/HIE synergy model. As in the spinal cord, histone deimination suggested a role for epigenetic regulation in response to injury, while changes in deimination of cytoskeletal components could affect apoptosis, cell motility and the ability of injured neurons to regrow axons (Lange *et al.* 2011), we focused on detecting changes in total protein deimination, and specifically deiminated histones, in response to PAD inhibition in the HIE model. The identification of target molecules for drug-directed early intervention in response to hypoxic insult is of great importance in relation to the prevention of long-term damage caused by oxygen deprivation and infections in neonates.

## Materials and methods

### Animals

All animal experiments and care protocols were approved by the Home Office (Permit number 70/7173) and carried out according to the UK Animals (Scientific Procedures) Act 1986. The ARRIVE guidelines were followed. Operations were performed on C57/BI6 mice (Charles River, UK) at post-natal day 7 (P7), bred in house. HIE: Animals at P7 were anaesthetized with isofluorane (5% induction, 1.5% maintenance), the left common carotid artery permanently occluded with an 8/0 polypropylene suture and the wound closed with tissue glue. The mice were recovered at 36°C, returned to the dam for 2 h and then placed in a hypoxia chamber and exposed to humidified 8% oxygen/92% nitrogen (2 L/min) at 36°C for 30 or 60 min. The mice were returned to the dam and left for 2, 4, 6, 8, 16, 24, 48 and 96 h, respectively, for post-hypoxia survival. In the infection/HIE synergy model, P6 pups were injected with *E. coli* lipopolysaccharide (LPS; 0.3 μg/g) 12 h prior to surgery.

### Tissue sample preparation

For histological assessment, animals were killed by intraperitoneal (i.p.) injection of pentabarbitone and perfused with 30 mL of phosphate-buffered saline (PBS). The brains were excised, post-fixed by rotating immersion in 4% formaldehyde in PBS for 1 h at 4°C, followed by cryoprotection for 24 h in 30% sucrose in PBS at 4°C (Moller *et al.* 1996). Fixed cryoprotected brains were frozen on dry ice, sectioned on a cryostat into sequential 40-μm sections and stored at −80°C until required.

### Pharmacological manipulation

#### Cl-amidine toxicity

Cl-amidine, a pan-PAD inhibitor (Luo *et al.* 2006) was dissolved in sterile normal saline and the animals injected with a single i.p. injection at dose of 30, 60 or 120 mg/kg (based on a previous study in spinal cord damage; Lange *et al.* 2011) immediately following 60-min hypoxia. Survival was assessed after 24, 48 and 72 h.

#### Pharmacological pan-PAD inhibition

To investigate the effect of PAD inhibition on tissue damage in the affected brain areas, animals were injected with a single i.p. dose of Cl-amidine (30 or 60 mg/kg; *n* = 5 per group; based on Lange *et al.* 2011) immediately after a time period of 60-min hypoxia. The Cl-amidine was diluted in saline so that all animals received a single 10 μL/g injection; control animals received the corresponding volume of saline. To estimate an effect on the synergy of infection and hypoxic ischaemic insult, Cl-amidine-treated LPS sensitized animals (0.3 μg/g) were injected 12 h prior to surgery with LPS (Kendall *et al.* 2011b), followed by one dose of Cl-amidine (30 mg/kg) 10 min after the LPS injection (*n* = 10 per group). A second dose of Cl-amidine (30 mg/kg) was administered immediately after a time period of 30-min hypoxia. Control LPS-treated animals received the corresponding amount of saline in substitution for Cl-amidine. The animals were left for 48 h, then sacrificed and brains collected for tissue analysis.

### Histological analysis

The differences in deiminated proteins, microglial activation, infarct size and cell death were compared between control animals (sham-operated control, saline treatment alone; LPS/saline treatment) and the corresponding PAD inhibitor-treated animals (Sal/Cl-amidine; LPS/Cl-amidine). All tissue sections were scored blindly twice by two independent observers.

### Immunohistochemistry

Tissue staining was performed as previously described (Hristova *et al.* 2010). In brief, cryosections were thawed and rehydrated in distilled water, spread onto glass slides coated with 0.5% gelatin under a dissecting microscope, dried for 10 min, fixed in 4% formaldehyde in 100 mM phosphate buffer (PB) for 5 min, treated with acetone (50, 100, 50%: 2 min each), 0.1% bovine serum albumin (PB/BSA) and washed twice in PB. The sections were pre-incubated with 5% goat serum (Sigma, St. Louis, MO, USA) in PB for 30 min and incubated with primary antibody overnight at 4°C (F95 (Nicholas and Whitaker 2002) 1/500; αMβ2 (Serotec, Oxford, UK) 1/5000; citH3 (Abcam, Cambridge, UK) 1/300). The sections were then washed in PB/BSA, PB, PB, PB/BSA (2 min each), incubated with secondary antibodies [biotin-labelled anti-mouse IgM; anti-rabbit IgG 1/200; anti-rat IgG (Vector Laboratories, Inc., Burlingame, CA, USA)] and visualized with Avidin-Biotinylated peroxidase Complex (ABC, Vector Laboratories, Inc.) and diaminobenzidine/hydrogen peroxide stain. Sections were processed through alcohol and xylene and mounted with DEPEX (Sigma). For quantitative immunohistochemistry, sections belonging to the same experiment were stained together to prevent differences in staining intensity.

### Infarct volume measurement

Infarct volume was measured in 10 coronal sections at 400-μm intervals from each forebrain, stained with cresyl violet (Nissl stain). The Optimas 6.2 image analysis software (Meyer Instruments Inc., Houston, TX, USA) was used to calculate the surviving brain tissue in each brain region as percentage between experimental and control side to estimate reduction in infarct size following PAD inhibition. Tissue injury score was calculated from the cresyl violet-stained sections (Nissl) and sections stained for activated microglia (αMβ2) as previously described (Kendall *et al.* 2006). The injury score was estimated on a scale from 0 to 4 for Nissl (0 = no damage, 1 = minimal evidence of damage without evidence of infarct, 2 = small infarct < 50% of the affected region, 3 = large infarct > 50% of the affected region, 4 = total neuronal loss). Score for microglial activation was on the scale from 0 to 3 (0 = no activation, 1 = focal activation, 2 = mild diffuse activation with occasional phagocytic macrophages, 3 = widespread activation with predominant phagocytic macrophages).

### TUNEL staining

Brain tissue sections were stained at 400-μm intervals for DNA fragmentation using Terminal deoxynucleotidyl transferase dUTP nick end labelling according to the manufacturer's instructions (TUNEL, Vectorlabs). Cell death was quantified by counting TUNEL-positive nuclei in each brain region and compared in treated versus control groups.

### Statistics

Statistical analysis to assess the effect of treatment (PAD inhibitor) proceeded in the following way. For each of the three outcomes, tissue loss, cell death and microglial activation, linear mixed effects models were fitted (using transformed data as necessary to satisfy normality assumptions), to adjust for the correlation between observations from the same subject arising from measurements in several areas of the brain (repeated measurements). Estimation of fixed effects was carried out using restricted maximum likelihood owing to the small sample size and tested using Wald tests. An interaction effect between the treatment group and the area of the brain was initially included in the model for each outcome and tested for significance. This tests whether the treatment difference is significantly different in different areas of the brain, although this test has low power to detect such a difference with small sample sizes. For all three outcomes the interaction was not significant, therefore main effects mixed models were fitted as the primary analysis. This gave an overall estimate of the treatment effect for each outcome with associated *p*-value related to testing the null hypothesis of no difference between treatment groups. If the treatment effect was significantly different from zero, (*p* < 0.05) further *post hoc* analysis was carried out to examine subregion treatment effects. This was done by refitting the model with the interaction to give estimates and associated *p*-values for the treatment effect in each area of the brain. It was done in this way rather than fitting separate regression models for each area to benefit from the properties of the estimation procedure used in mixed effects models that help retain the validity of the results in the presence of incomplete data. No adjustments are made for multiple comparisons as actual *p*-values are reported.

## Results

### Protein deimination in neonatal brain following HI insult

**Fig 1:**
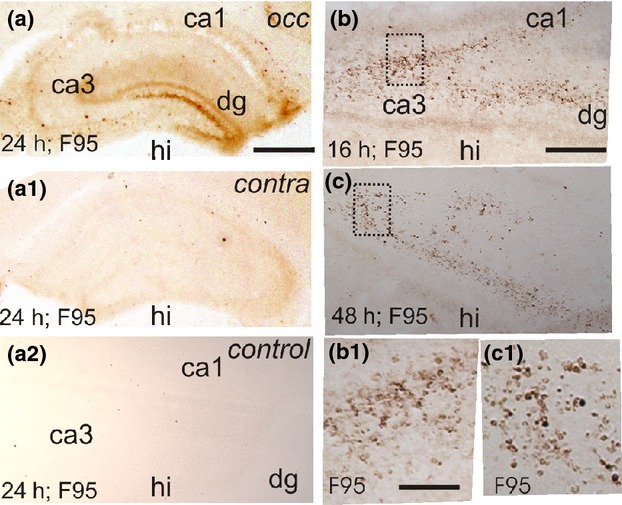
Protein deimination in neonatal hypoxic ischaemic (HI) hippocampus. Strong increase in hippocampal (hi) pan-peptidyl citrulline immunoreactivity detected with F95 antibody on the occluded (*occ*) side (a), compared with the contralateral (*contra*) (a1), after unilateral carotid ligation in post-natal day 7 mice, 60-min exposure to 8% oxygen (strong hypoxia) and 24-h recovery. Note the intense staining in the dentate gyrus (dg) molecular layer and around the granular cell band, as well as scattered, highly immunoreactive cells from the hilus to the CA1 region and in the CA3 region. No protein deimination is observed in sham-operated controls (b2; hippocampus at 24-h recovery). Protein deimination is clearly detectable earlier at 16 h post insult (b: b1) and still present at 48 h post insult when clear tissue degradation in the damaged region is also observed (c; c1). b1 and c1 represent magnified images of the dotted boxes indicated in the CA3 regions in b and c respectively. Scale bars: a–a2 = 500 μM; b–c = 200 μM; b1–c1 = 100 μM.

Total protein deimination detected with the pan-citrulline antibody F95 (Nicholas and Whitaker 2002) showed that protein deimination (F95 positive) started at 16 h, peaked at 24 h and was still detectable at 48–72 h following mild (30 min) HI insult. Following strong (60 min) hypoxia, deiminated proteins were detected at 8 h, increasing and peaking at 16–24 h (Fig.1a,b, b1) and still strongly detectable at 48–72 h (Fig.1c & c1). Deiminated proteins were mainly detected in the hippocampus, cortex, striatum and piriform cortex. Fig.1 shows the several-fold increase in total protein deimination (F95 positive) observed in the hippocampus (hi) of the occluded side (Fig.1a–c, b1 & c1) following 60-min exposure to 8% oxygen compared with the non-occluded side (Fig.1a1). No protein deimination was observed in the comparable regions in sham-operated controls (Fig.1a2).

### Deiminated protein targets in the HI/infection synergy model

**Fig 2:**
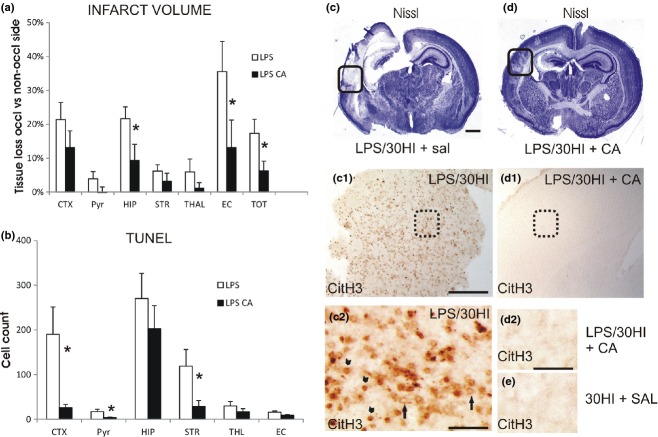
Peptidylarginine deiminase (PAD) inhibition reduces neuronal loss and histone deimination. PAD inhibition results in reduced infarct volume (a) and cell death (b) 48 h following lipopolysaccharide (LPS) sensitized hypoxic ischaemic (HI) (LPS/HI) insult (*n* = 10 per group). (a) For tissue loss, significant differences were seen in the overall PAD inhibition [Cl-amidine (CA)] treatment (*p* = 0.007), with some *post hoc* evidence of subregion differences in the hippocampus and external capsule (*p* = 0.059 and *p* = 0.013 respectively) (b) Following PAD inhibition (CA) cell death was significantly reduced overall (*p* < 0.001), with *post hoc* evidence of subregion reduction in cortex, pyriform cortex and striatum (*p* < 0.001, *p* = 0.019, *p* = 0.003), with some *post hoc* evidence of an effect in hippocampus and thalamus (*p* = 0.062, *p* = 0.058). (c, d) Nissl-stained brains of the LPS/HI animals post-treated with saline (sal) show massive tissue loss on the occluded side (left) in the cortex, hippocampus, striatum and external capsule (c); those post-treated with Cl-amidine (CA, 1 × 30 μg/g 10 min after LPS injection and again 1 × 30 μg/g immediately after 30-min hypoxia) result in a greatly reduced infarct (d). c1 (boxed region in c): Massive increase in deiminated histone 3 immunoreactivity (citH3) is observed 48 h following combined LPS/HI insult, and post-treatment with saline. d1 (boxed region in d): CitH3 is suppressed in LPS/HI animals treated with Cl-Amidine. c2 (higher magnification of c1): Histone deimination is observed in the nucleus (arrowheads) and cellular cytoplasm (arrows) in the damaged cerebral cortex in LPS/HI animals. d2 (higher magnification of d1): Both types of citH3 immunoreactivity are suppressed in LPS/HI animals treated with Cl-amidine and absent in control 30 min HI alone (e). Scale bars: c & d: 1 mm; c & d1 = 250 μM; c,2 d2 & e = 100 μM; *p < 0.02.

Using the infection/HI synergy model by pre-exposing day 6 mouse pups to *E. coli* LPS, followed 12 h later by unilateral carotid occlusion and 30 min of 8% oxygen, an overall increase in pan-deiminated proteins was observed compared to HI insult alone (not shown). Markedly, a massive increase in deiminated histones (citH3), a target of the PAD4 isozyme, was observed in the affected brain regions upon exposure to HI/LPS (30 min HI; Fig.2c1 & c2) but was not observed in the control mild HI insult alone (30 min HI; Fig.2e).

### PAD inhibition reduces neuronal damage in HI and HI/infection synergy models

**Fig 3:**
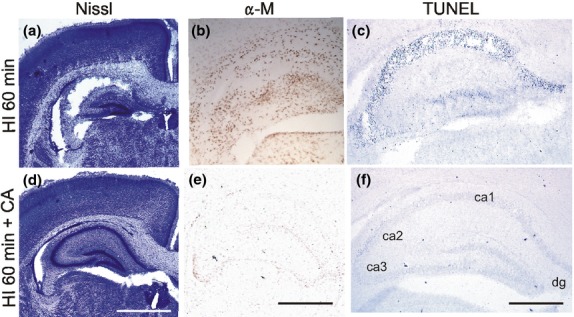
Peptidylarginine deiminase (PAD) inhibition reduces neuronal damage in neonatal hypoxic ischaemic (HI) with 60-min (strong) hypoxia. Following HI with strong (60 min) hypoxia, severe tissue loss (a), strong microglial activation with phagocytic morphology (b) and TUNEL-positive cells (c) were observed in the hippocampus after 48 h. Upon treatment with one dose of Cl-amidine (CA; 60 mg/kg) immediately following hypoxia, reduced damage was seen in the hippocampus (d) and a significant difference was observed both in the level of microglial activation (e) and TUNEL-positive cells (f). Scale bars: a & d = 500 μM; b, c, e, f = 200 μM. For reference, the regions of the hippocampus (dentate gyrus (dg) and CA 1, 2, 3) are indicated in f.

**Fig 4:**
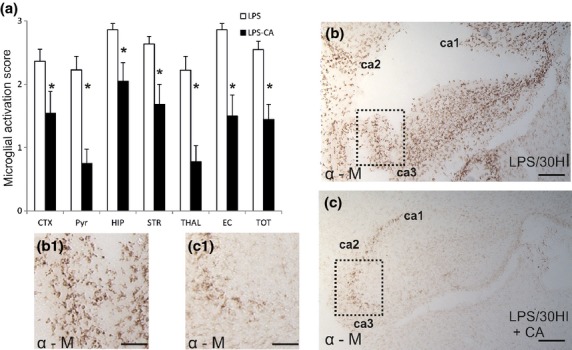
Peptidylarginine deiminase (PAD) inhibition reduces microglial activation in the neonatal lipopolysaccharide (LPS)/hypoxic ischaemic (HI) synergy model. For microglial activation (α − M), the overall adjusted treatment effect (CA) was significant (*p* < 0.001), and *post hoc* evidence of subregion differences were found in the cortex, pyriform cortex, hippocampus, striatum, thalamus and external capsule (*p* = 0.012, *p* < 0.001, *p* = 0.017, *p* = 0.003, *p* < 0.001, *p* < 0.001 respectively). Whereas LPS-treated control brains show strong microglial activation with phagocytic morphology (b & b1: hippocampus), Cl-amidine (CA)-treated brains show only focal activation of microglia (c & c1: hippocampus: CA 1, 2, 3 regions). b1 and c1 are magnified images from the boxed regions of CA3 in b and c respectively. Scale bars: b & c = 200 μm, b & c = 100 μm; *p < 0.02.

The effects of the pan-PAD inhibitor Cl-amidine (Luo *et al.* 2006) on 48-h survival of 7-day-old mice in the HI insult model were estimated to determine the optimal treatment dose. Intraperitoneal application of Cl-amidine at doses of 30 mg/kg and 60 mg/kg were associated with 100% survival; and 120 mg/kg with 75% survival. By itself carotid occlusion and oxygen starvation was not associated with a 48-h loss. Treatment with Cl-amidine at 60 mg/kg was determined for further use as it gave maximum inhibition of damage (compared to 30 mg/kg) and 100% survival. Using 60 mg/kg Cl-amidine single dose, neuronal loss, cell death and microglial activation were reduced in response to PAD inhibition in HI alone following 60-min hypoxia (Fig.3a–f; *n* = 5). In the LPS/HI synergy model, Cl-amidine was administered in 2 doses of 30 mg/kg each to inhibit PAD after LPS stimulation (10 min post stimulation) and again after HI insult (straight after 30-min hypoxia), a total of 60 mg/kg in a 24-h period, resulting in significantly decreased neuronal tissue loss and cell death (Fig.2a–d). For tissue loss, significant differences were seen in the overall treatment (*p* = 0.007), with some *post hoc* evidence of subregion differences in the hippocampus and external capsule (*p* = 0.059 and *p* = 0.013 respectively) (Fig.2a). Cell death was significantly reduced overall (*p* < 0.001), with *post hoc* evidence of subregion differences in cortex, pyriform cortex and striatum (*p* < 0.001, *p* = 0.019, *p* = 0.003), with some *post hoc* evidence of an effect in hippocampus and thalamus (*p* = 0.062, *p* = 0.058) (Fig.2b). The presence of deiminated histones was drastically reduced when applying Cl-amidine in the LPS synergy model (Fig.2d1–d2) compared with LPS-stimulated groups (Fig.2c1–c2). For microglial activation (α − M), the overall adjusted treatment effect (CA) was significant (*p* < 0.001), and *post hoc* evidence of subregion differences was found in the cortex, pyriform cortex, hippocampus, striatum, thalamus and external capsule (*p* = 0.012, *p* < 0.001, *p* = 0.017, *p* = 0.003, *p* < 0.001, *p* < 0.001 respectively) (Fig.4a). Moreover, while strong microglial activation with phagocytic morphology was observed in the LPS/Saline-treated brains (Fig.4b & b1), microglia displayed only focal activation in the presence of the PAD inhibitior (Fig.4c & c1).

## Discussion

We have shown that protein deimination caused by peptidylarginine deiminases (PADs) plays a significant role in neuronal loss following hypoxic ischaemic insult in the neonatal mouse model and that pharmacological PAD inhibition may be useful to reduce neuronal loss when applied following hypoxia. Histones are one of the major targets of deimination, and PAD4-mediated histone 3 deimination (citH3) is associated with both gene regulation and the formation of neutrophil extracellular traps in response to infection (Li *et al.* 2010). We found that histone 3 is significantly deiminated specifically upon stimulation with LPS, as non-stimulated HI-treated control animals showed very low or no histone deimination (Fig.2E). Upon Cl-amidine treatment, citH3 detection was drastically reduced in LPS-stimulated animals and similar to that seen in non-stimulated animals (30 min HI; Fig.2d2 & e). The strong up-regulation of citH3 in the LPS synergy model, and the corresponding reduction of histone deimination following PAD inhibition, indicates a possible epigenetic role for PAD4. In terms of injury signals, the LPS/HI model of infectious/ischaemic form of brain damage is known to be strongly dependent on TNFα and related family of cytokines. Global gene deletion of the whole TNF cluster of cytokines – TNFα, LTα, LTβ – has been shown to completely abolish the synergistic, damage-enhancing effect of LPS on HI insult (Kendall *et al.* 2011b). Exposure to elevated levels of TNFα has also been demonstrated to elicit nuclear translocation of PAD4 isozyme *in vitro*, as well as *in vivo*, in a transgenic model of multiple sclerosis (Mastronardi *et al.* 2006). Here, we show that broad pharmacological inhibition with the pan-PAD inhibitor Cl-amidine in the LPS/HI model causes a significant reduction in infarct size, microglial activation and TUNEL+ cell death compared to the control, LPS-treated animals (Figs2 and 4).

Overall, the data presented here support our hypothesis that PAD activity is induced both in HI insult alone and in HI insult combined with LPS stimulation. A more selective and targeted inhibition of individual PAD enzymes, than demonstrated here using a pan-PAD inhibitor, could lead to enhanced neuroprotection following HI insult, as well as help uncover the downstream targets of the neuro-destructive proinflammatory cytokines. It has been shown, for example, that TNFα appears to specifically affect PAD4 (Mastronardi *et al.* 2006). The peak in TUNEL-positive cells being 8–16 h for the 30 min HI and 16–24 h for the 60-min hypoxia, respectively, shows that protein deimination in the strong HI insult precedes detection of apoptotic cell death, while it coincides in the milder (30 min) hypoxic conditions. This may indicate a stronger involvement of PADs in cell death under strong hypoxic conditions. The present findings are in accordance with a previous study in the spinal cord injury model where pan-PAD inhibition resulted in significantly reduced injury volume, cell death and citH3 detection (Lange *et al.* 2011), indicating that protein deimination plays an important role in neuronal damage.

The neuroprotective effect we have demonstrated here using pan-PAD inhibiton provides a platform for refined isozyme-specific drug development for targeted intervention in events of neonatal HIE. Our findings may be translatable to other forms of neuronal damage and benefit intervention in those cases. Ongoing studies aim to identify in depth the respective PAD isozymes and target proteins for drug-directed treatment in neonatal HIE. Novel drugs targeting the appropriate PAD isozyme may be new candidates for the prevention of neural impairment caused by oxygen lack and infection in neonates.

## Acknowledgments and conflict of interest disclosure

The study was supported in part by grants from Wellbeing of Women (RG1266) and the UCL Graduate School. The authors declare no competing financial interests.

All experiments were conducted in compliance with the ARRIVE guidelines.

## Abbreviations

BSA: bovine serum albumin
HIE: HI encephalopathy
HI: hypoxic ischaemic
PBS: phosphate-buffered saline
